# Evaluating the Role of Mortar Composition on the Cyclic Behavior of Unreinforced Masonry Shear Walls

**DOI:** 10.3390/ma17184443

**Published:** 2024-09-10

**Authors:** Meera Ramesh, Rafael Ramirez, Miguel Azenha, Paulo B. Lourenço

**Affiliations:** 1 Ryan Biggs Clark Davis, New York, NY 12065, USA; 2 Department of Civil Engineering, University of Minho, ISISE, ARISE, 4800-058 Guimarães, Portugal

**Keywords:** lime-cement mortar, brick masonry, in-plane cyclic loading, digital image correlation, drift capacity, stiffness degradation, energy dissipation, shear capacity

## Abstract

The mechanical behavior of unreinforced masonry (URM) shear walls under in-plane cyclic loading is crucial for assessing their seismic performance. Although masonry structures have been extensively studied, the specific influence of varying lime content in cement-lime mortars on the cyclic behavior of URM walls has not been adequately explored. This study addresses this gap by experimentally evaluating the effects of three mortar mixes with increasing lime content, 1:0:5, 1:1:6, and 1:2:9 (cement:lime:sand, by volume), on the cyclic performance of brick URM walls. Nine single-leaf wall specimens 900 mm × 900 mm were constructed and subjected to combined vertical compression and horizontal cyclic loading. Key parameters such as drift capacity, stiffness degradation, and energy dissipation were measured. The results indicated that the inclusion of lime leads to a moderate improvement in drift capacity and ductility of the walls, with the 1:1:6 mix showing the highest lateral capacity (0.55 MPa), drift at cracking (0.08%), and drift at peak capacity (0.31%). Stiffness degradation and energy dissipation were found to be comparable across all mortar types. These findings suggest that partial substitution of cement with lime can enhance certain aspects of masonry performance. Further research is recommended to optimize mortar compositions for unreinforced masonry applications.

## 1. Introduction

Masonry tends to fail in shear when exposed to both vertical and horizontal loads, as usually occurs during earthquakes [1]. There are two main approaches to studying the response or masonry to this type of loading, namely quasi-static cyclic tests, and dynamic shaking table tests [2]. Quasi-static cyclic tests are better suited for testing unreinforced masonry since they allow for more precise measurements of damage, whereas dynamic tests simulate seismic forces with greater accuracy [3]. However, quasi-static cyclic tests are considered more conservative because they often result in greater damage and lower lateral capacity in the specimens compared to dynamic tests [2]. Although lateral strength capacity is an important parameter, other factors such as strength and stiffness degradation, energy dissipation, and ductility are also necessary to evaluate the response of an unreinforced masonry structure to seismic loads. Higher ductility in masonry enhances the capacity for non-linear deformation, which leads to improved seismic performance [2]. Generally, deformations are compared using lateral drift, which is the ratio of lateral displacement to the wall height, expressed as a percentage [2]. Energy dissipation is also essential in evaluating seismic performance, as it involves a potential reduction in the demand for ductility [4]. In this context, cyclic tests are proposed to simulate the alternating loading direction typical of earthquakes [5,6,7,8].

**Figure 1:**
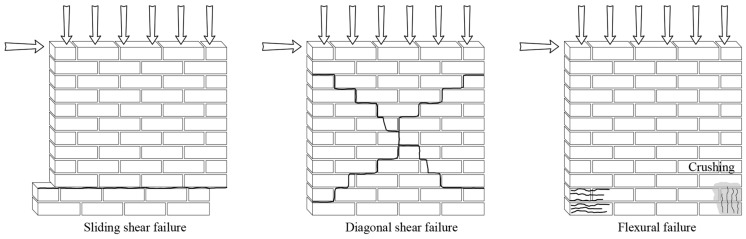
Typical failure modes of masonry walls subjected to combined in-plane vertical and horizontal loads.

The in-plane behavior of unreinforced masonry walls depends on various factors, including geometry, type of bond, vertical loading, boundary conditions, and mechanical characteristics of its constituents [1]. Moreover, different failure modes may occur, depending on the vertical loading, quality of bond between the unit and mortar, as well as bond strength (Figure 1) [9].

The first type of failure is referred to as sliding failure and is associated with low vertical stresses and poor-quality mortar. This results in the wall splitting into two parts, which slide over each other [10]. This type of failure is stable and leads to large deformations and energy dissipation [1,10]. The second type of failure, known as diagonal shear failure, usually happens when in-plane principal stresses surpass the tensile strength of masonry [10]. This results in diagonal cracking, low deformation capacity, and relatively fast degradation of strength and stiffness, with average energy dissipation. The quality of individual components determines whether cracks form through the units, the mortar joints, or both [1]. The third failure mode, known as rocking-flexural failure, is generally associated with slender walls with a high moment-to-shear ratio, low vertical stresses, high deformation capacity, and average energy dissipation, with scarce strength degradation. This type of failure also involves masonry crushing under compression at the corners [10].

During cyclic tests, flexural mechanisms tend to develop initially due to the low tensile capacity of masonry, which can be mistaken for the final failure mode [1]. Indeed, horizontal tensile cracks usually appear near the supports, sometimes with corner crushing, but the capacity of the walls generally increases until the eventual shear failure occurs. Notably, the sliding failure mode is controlled by shear bond strength, which is dependent on the cohesion and friction between masonry units and mortar joints. Conversely, the diagonal failure mechanism is controlled by the principal tensile strength [1,11] and can be expressed analytically by assuming elastic, isotropic behavior of the masonry until failure [11,12], such as:

(1) $$f_{t}=\sqrt{{\left(\frac{\sigma }{2}\right)}^{2}+{\left(r\;\tau_{max}\right)}^{2}}-\frac{\sigma }{2}$$

where σ [Pa] is the vertical compressive stress, r [-] is a dimensionless factor that considers the wall height-to-length ratio, and $\tau_{max}$ [Pa] is the shear stress corresponding to the maximum lateral load capacity of the wall.

Unreinforced masonry walls can fail through the development of diagonal cracks, either passing through the mortar joints, the units, or both [13,14,15]. However, the wall can still have some bearing capacity after the formation of these cracks if the units and mortar are of good quality [16]. Different analytical methods have been proposed to estimate the shear capacity of masonry under cyclic loading, depending on the observed failure modes [11]. In general, these analytical expressions sacrifice accuracy in favor of simplicity, but they are equally valid in identifying and evaluating the most important factors that contribute to the shear behavior of unreinforced masonry walls [3]. As an example, the Mohr–Coulomb law is recommended by Eurocode 6 [17] to determine the design shear force:

(2) $$V_{d}=\frac{\left(f_{vk0}+\mu \;\sigma \right)\;t\;l}{\;\gamma_{m}}$$

where $f_{vk0}$ [Pa] is the initial shear strength of masonry, defined by the type of mortar and its strength class, *μ* = 0.4 is the friction coefficient, $\gamma_{m}$ [-] is the safety coefficient for the material, and t [m] and l [m] are the thickness and length of the wall, respectively. However, it has been argued that Equation (2) is only valid to estimate pure sliding shear failure since it disregards any contribution of the tensile strength of the material [6].

Other expressions have been presented to estimate the shear resistance of masonry depending on failure mode, such as the approach proposed by Magenes & Calvi [18], applicable for cases with failure through mortar joints:

(3) $$V_{d}=t\;l\;\tau_{u}\;\mathrm{where}\;\tau_{u}=\min\left(\tau_{c},\;\tau_{w}\right)$$

$$\tau_{c}=\frac{1.5\;c+\mu \;\sigma }{1+3\;c\;\alpha_{V}/\sigma }\;\;\mathrm{relevant}\;\mathrm{to}\;\mathrm{the}\;\mathrm{cracked}\;\mathrm{section}$$

$$\tau_{w}=\frac{c+\mu \;\sigma }{1+\alpha_{V}}\;\;\mathrm{relevant}\;\mathrm{to}\;\mathrm{the}\;\mathrm{whole}\;\mathrm{section}$$

where $\tau_{u}$ [Pa] is the ultimate shear strength, c [Pa] represents cohesion, and $\alpha_{V}=h_{0}/l$ is the ratio defined by the height and the length of the wall, $h_{0}$ [m] and l [m], respectively.

Alternatively, Mann & Mueller [19] proposed the following expression to account for failure associated with shear-tensile cracking of masonry units (bricks):

(4) $$V_{d}=t\;l\;\frac{f_{tb}}{2.3\;\left(1+\alpha_{v}\right)}\sqrt{1+\frac{\sigma }{f_{tb}}}$$

where $f_{tb}$ [Pa] is the tensile strength of the brick.

For rocking-flexural failure, Magenes & Calvi [18] proposed the following analytic expression based on equilibrium:

(5) $$V_{d}=\frac{t\;l\;\sigma }{2\;\alpha_{v}}\left(1-\frac{\sigma }{j\;f_{u}}\right)$$

where j= 0.85 accounts for the distribution of vertical stresses at the compressed toe, assuming an equivalent rectangular stress distribution.

While numerous studies have explored the properties of lime-cement mortars at a material level, there has been limited systematic research specifically focused on the influence of lime content in these mortars when used in unreinforced brick masonry. Although recent research has focused on the mechanical characterization of masonry built with lime-cement mortars in different proportions, e.g., [20,21,22,23], these studies generally do not address their behavior under in-plane cyclic loading. The lack of in-depth analysis in this area leaves a gap in understanding how varying lime content within these mortars can affect the structural performance of unreinforced masonry, particularly under lateral loads such as the ones imposed by seismic actions.

On the other hand, extensive research has been conducted using in-plane cyclic tests to evaluate the performance of masonry walls. In particular, a considerable number of studies have been devoted to studying how different reinforcement techniques, such as fiber-reinforced polymers (FRP) and textile-reinforced mortars (TRM), can improve the masonry lateral strength and its capacity to dissipate energy when subjected to cyclic loads [13,15,24,25,26,27,28,29,30,31,32]. Other studies have focused on the impact of boundary conditions or the presence of wall openings on the in-plane cyclic response of masonry walls [28,29,33,34,35,36]. Moreover, the response of stone masonry walls to in-plane cyclic loading has been investigated as a function of their textural typologies, concluding that ductility decreases as the irregularity of bonds increases [37]. In a study of unreinforced masonry with lime-based mortar, the performance of walls built with three different lime mixes (B/Ag ratios 1:1, 1:2, 1:3) was compared for quasi-static cyclic loading, and it was observed that the stiffness and energy dissipation capacity increased proportionally to the compressive strength of the mortar, while ductility followed the opposite trend [14]. The maximum drift capacity reported in the literature for fired-clay brick unreinforced masonry walls ranges between 0.43% and 1.06%, with average of 0.60% [38]. Moreover, the maximum drift capacity decreases with increasing vertical loads, indicating an inverse relationship with respect to the compressive stress state [2,38]. The maximum lateral capacity values of fired-clay brick unreinforced masonry walls found in the literature range between 0.3 MPa and 0.5 MPa [1], but specific research on the impact of lime-cement mortars on this aspect could not be found.

Despite these efforts, there remains a significant gap in the literature regarding the systematic study of how varying lime content in masonry mortars influences the in-plane cyclic shear response of unreinforced brick masonry walls. Addressing this gap, this article focuses on the experimental investigation of unreinforced masonry walls built with three different mortars 1:0:5, 1:1:6, and 1:2:9 (cement:lime:sand, by volume) and solid frogged fired-clay bricks. By subjecting these masonry specimens to quasi-static cyclic loading, we aim to systematically assess how varying lime content affects their in-plane shear response.

## 2. Materials and Specimens

The binders chosen to prepare the mortar mixes were Portland cement and air lime. CEM I-42.5 R was selected as the cement binder to minimize material variability. CEM I is known for its lower allowable variation in constituent materials compared to other cement types, such as CEM II [39]. This characteristic is particularly important in experimental studies where consistency and reproducibility are crucial. The batch of cement used in this campaign was supplied by the manufacturer SECIL (Setubal, Portugal). The chemical composition of the cement binder, as specified by the manufacturer, is presented in Table 1. According to the technical data sheet, the material has a Blaine-specific surface of 3508 cm^2^/g, and a clinker composition of 12.6% C2S and 62.2% C3S. The apparent bulk density of cement measured in the laboratory was 0.93 g/cm^3^. Conversely, the type of lime chosen for the mortar mixes was CL 90-S. As mentioned for cement, the selection of CL 90-S was performed with the scope of minimizing material variability, considering that CL 90-S presents the least variation in chemical composition as well as the highest amount of available lime in comparison to other air lime types [40]. The lime was supplied by Lhoist (Valverde, Portugal), and following their technical data sheet, the density was 2.24 g/cm^3^, whereas the BET-specific surface was 150,000 cm^2^/g. Moreover, the particle size distribution presented a mean value between 5.5 µm and 6.5 µm. Further details on the chemical composition of the lime binder are presented in Table 1. In the table, LOI stands for loss on ignition and is a value measured by the manufacturer according to EN 459-2 [41]. Finally, the apparent bulk density determined in the laboratory was 0.36 g/cm^3^.

The selection of the masonry unit was made by considering various options available in the market [42,43,44], including concrete blocks, autoclave aerated concrete blocks, calcium silicate bricks, and fired-clay bricks. Moreover, the choice of the unit for the construction of the walls was based on information from the literature [43,45,46,47,48,49], as well as on the opinion of experts from the industry [50]. The evaluation of clay bricks was performed based on their geometry, presence of voids (solid, perforated, or hollow), and manufacturing process, including molded or extruded bricks [51]. Solid molded fired-clay bricks were ultimately chosen based on their water absorption properties and initial rate of absorption (IRA). In this sense, it was assumed that bricks with higher water absorption and IRA could better reveal the differences in the mechanical performance of masonry according to the type of binder used in the mortars. Therefore, a solid molded fired-clay brick with frogs, manufactured by Wienerberger, was selected. The brick has dimensions 215 mm × 102 mm × 65 mm, and according to standard EN 771-1 [51], it falls into the group 1 category, with tolerance T1, range R1, and volume of frogs below 20%.

The brick was experimentally tested for water absorption, initial rate of absorption (IRA), compressive strength, flexural strength, and Young’s modulus. The corresponding results are shown in Table 2, along with their respective scatter expressed as the coefficient of variation (CoV). The mean compressive strength of the brick was 26.2 MPa. Following the recommendations of EN 772-1 [52], the average compressive value was multiplied by a shape factor to determine the normalized compressive strength. In the present study, the shape factor varied between 0.55 and 0.66 depending on the specific height-to-width ratio of each specimen. These variations were found to lie within the acceptable ranges of the tolerance category T1 of EN 771-1 [51]. The average value of the normalized compressive strength is presented in Table 2.

Three types of mortars were selected with varying compositions of binders and aggregates, namely 1:0:5 (REF), 1:1:6 (L50), and 1:2:9 (L67) (cement:lime:sand, expressed by volume) (see Table 3). It is noted that the designation ‘L’ represents the percentage of lime in the binder. These specific mortar proportions were chosen to align with those used in previous studies [23,53], where they were identified as representative of mortar mixes commonly used in the field. This approach ensures continuity in research and relevance to practical applications. To further ensure the practical use of these mortars by masons in real applications, the water–binder ratios for all the mixes were chosen so that their flow table value resulted in 175 ± 10 mm.

In total, nine masonry wall specimens were prepared for cyclic load testing, with three walls prepared for each of the three different mortar mixes. The masonry specimens were built with 10 mm thick mortar joints [54,55,56,57,58,59]. Controlled laboratory conditions, 21 ± 1 °C and 60 ± 5% RH were maintained during the construction, curing, and testing of the specimens. Moreover, all the materials, including binders, sand, bricks, and water, were stored in the same conditions prior to the construction of the walls. Based on the recommendations of RILEM LUM B1 [60], all batches of mortar were employed within a span of 60 min following their preparation, taking the starting time as the instant when the binders were brought into contact with water. Approximately 13 kg of sand was used for every batch of mortar, ensuring that the complete mortar volume was utilized for the construction of the walls within the said timespan of 60 min. Measurements of all materials, including binders, sand, and water, were conducted by weight to increase accuracy in the mix design. The components were mixed in a barrel for 90 s, with the sand being introduced between 30 and 90 s. The mixing process was paused for 90 s, and during this time, the mortar was carefully scraped from the walls and the base of the barrel. The process concluded with another 90 s of mixing. The employed mixer was a representative example of what is commonly used by masons in practical applications, suitable for mixing large volumes. The specific model utilized was Parkside PFMR 1600 D5. According to the manufacturer, this mixer has a rotational speed of 800 revolutions per minute, a motor capacity of 1600 W, and a stirrer holder type M14 [61].

Before being used for masonry construction, the bricks were thoroughly cleaned and soaked in water for approximately 30 min. This was carried out to prevent the bricks from absorbing water from the mortar, which could adversely affect the bond strength of masonry [60]. The task of building the masonry specimens was undertaken by an experienced mason affiliated with the Structures Laboratory of the Civil Engineering Department at the University of Minho. Thus, the nine masonry specimens for in-plane cyclic tests were built within the span of one week. These specimens consisted of single-leaf walls with a running bond pattern, with 4 bricks in length and 12 courses in height, and overall dimensions 900 mm × 900 mm × 102 mm.

The mortars prepared for material characterization were cured under two different conditions for comparison purposes:

- Standard curing. The mixing procedures recommended by the European standards [62,63] were followed for the three mortar mixes, namely REF, L50, and L67. The cement-only mix (REF) was cured following the specifications of EN 196-1 [63]. Therefore, the molds containing this mix were covered with plastic and kept in a climatic chamber, 20 ± 1 °C and 95 ± 5% RH, for the first 24 h. After that, the REF specimens were demolded and submerged in water at 20 ± 1 °C until the time of testing. The other two mortar mixes containing lime-cement binders (L50 and L67), were cured in a climatic chamber, 20 ± 1 °C and 95 ± 5% RH, during the first 7 days, and then at 20 ± 1 °C and 65 ± 5% RH until the age of testing. The standard EN 1015-11 [62] was used to decide when to demold the lime-cement mortar specimens, recommending demolding after 2 days if the lime content in the binder is lower than 50% by mass.
- In situ conditions. The three types of mixes (REF, L50, and L67) employed for the construction of masonry specimens were cast in large quantities. During the preparation of the walls, small portions of mortar were taken from these large batches and placed into molds near the masonry specimens. Thus, these “in situ mortars” were cured under the same temperature and relative humidity conditions as the masonry walls.

The mortar specimens prepared under these two curing conditions were subjected to flexure and compression tests as specified in EN 1015-11 [62]. The results of the mechanical characterization tests on mortars are presented in Table 4. For further details on the mechanical properties of the mortars, the reader is referred to [53].

## 3. Experimental Setup

**Figure 2:**
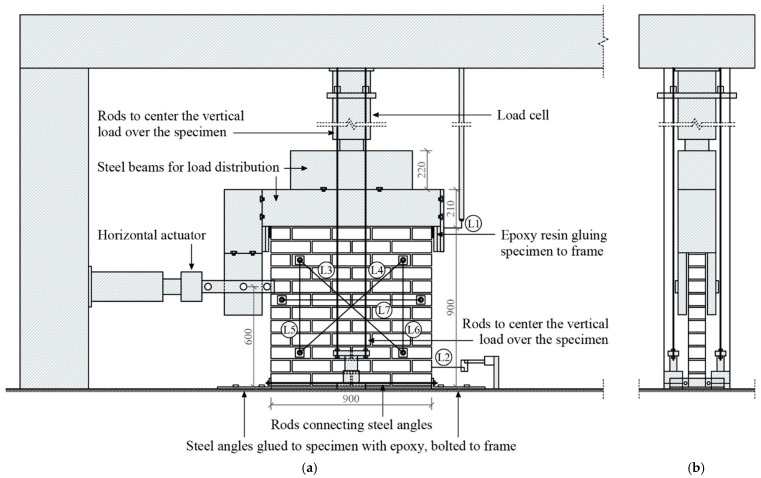
Experimental setup used in this study for in-plane cyclic loading tests of masonry specimens (L stands for LVDT): (**a**) front view; (**b**) lateral view. Dimensions in mm.

In-plane cyclic tests were performed on the masonry specimens described above, approximately 6 months after their construction. Prior to testing, the top and bottom faces of the specimens were leveled using a rapid hardening cement paste (Mapei Lampocem [64]) to ensure a uniform load distribution. For the rectification of the lower face, the specimens were lifted using a crane and two wide straps and then lowered onto a layer of freshly mixed cement. The specimens were lifted with two wide straps and a crane and then lowered onto a fresh layer of cement. After several hours, the upper face was rectified as well using the same cement paste, a metallic beam, and a level. Once the leveling process was finalized, each specimen was moved, using the crane and straps, to the frame where the test would be conducted. A schematic representation of the test setup is illustrated in Figure 2.

In order to fix the bottom of the specimen and prevent horizontal movement, steel angles (60 mm high, 300 mm wide, thickness of 20 mm) were glued on both ends of each specimen using polyester resin. Moreover, the steel angles were tied together by means of two steel rods ∅10 mm, one on each side of the specimen, and subsequently anchored to the base of the frame where the test was conducted. A vertical load of 72 kN, corresponding to a compressive stress of 0.78 MPa, was applied uniformly along the top face of the specimen using rigid steel beams. It is noted that this pre-compression level was selected because it entailed around 12% of the ultimate compressive strength of the masonry and was within the typical range of values found in the literature [2,65,66]. The actuator responsible for the vertical load was also secured to the base of the frame using four steel rods, which helped maintain the load centered on the specimen during the horizontal movement.

Horizontal displacements were imposed on the specimens using an actuator with a 200 kN capacity and sensitivity of 2 mV/V. The horizontal actuator was connected to a rigid vertical beam by means of a hinge. The vertical beam, in turn, was fixed to the horizontal rigid beams on top of the specimen. Moreover, the actuator was placed 600 mm above the base of the specimen. This height, together with the compression level, was chosen to guarantee shear failure. The deformations of the specimens during the test were measured using seven Linear Variable Differential Transformers (LVDTs) as illustrated in Figure 2. LVDT 1 (±300 mm measuring range), located on the top, was employed to measure the lateral displacement in the specimen, whereas LVDT 2 (±100 mm measuring range), located at the bottom, second course from the base, was used to monitor the potential development of flexural cracks. The remaining five LVDTs (diagonals with a measuring range of ±200 mm; horizontal and vertical ones with a range of ±100 mm) were placed on the rear face of the specimen to obtain additional deformation data.

Digital Image Correlation (DIC) was employed in this study due to its proven effectiveness in providing precise, full-field strain measurements and capturing the complex deformation behaviors in masonry structures under cyclic loading conditions, as demonstrated in recent studies on similar materials and loading scenarios [34,66,67,68]. The front face of the specimen was coated with a homogeneous layer of white paint and then a random pattern of matte black spray was manually applied. A professional photographic camera (Cannon EOS M50) was positioned on a tripod, approximately 1.30 m from the target wall surface, centered and perpendicular to it. The camera had an aperture setting of F 3.5 and a shutter speed of 1/40, and images were taken at 5 s intervals. The calibration of the DIC system was performed by placing an object with known dimensions in the same plane as the specimen, which allowed for precise scaling of displacement values. The open-source software GOM Correlate (v.2.0.1) was used to process the DIC results. In this study, DIC was used qualitatively to provide an indication of the principal strains that represent cracks in the masonry.

**Figure 3:**
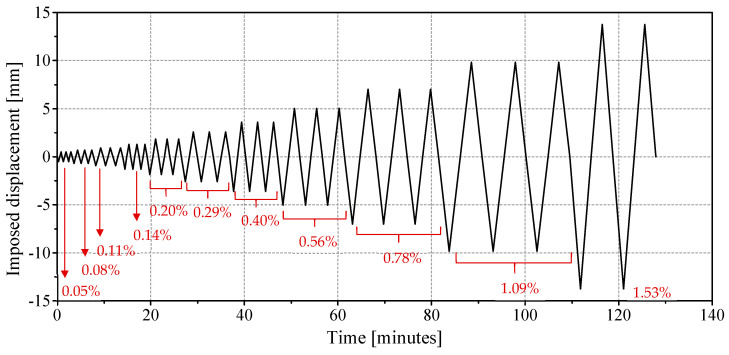
Horizontal deformations imposed on masonry specimens for in-plane cyclic loading test (drift in %).

Following the guidelines of FEMA-461 [69], the horizontal lever was used to apply horizontal displacements in a ramp format, as presented in Figure 3 and Table 5. The initial drift was set at 0.05%, whereas the maximum drift was defined as 1.5% (if failure had not occurred before). The amplitude increased after the cycle had repeated three times, at a rate of 1.4 times the value of the previous level. The speed of the displacements was adjusted during the test for each amplitude level in order to minimize dynamic effects and ensure that the test duration was reasonable.

## 4. Results and Discussion

**Figure 4:**
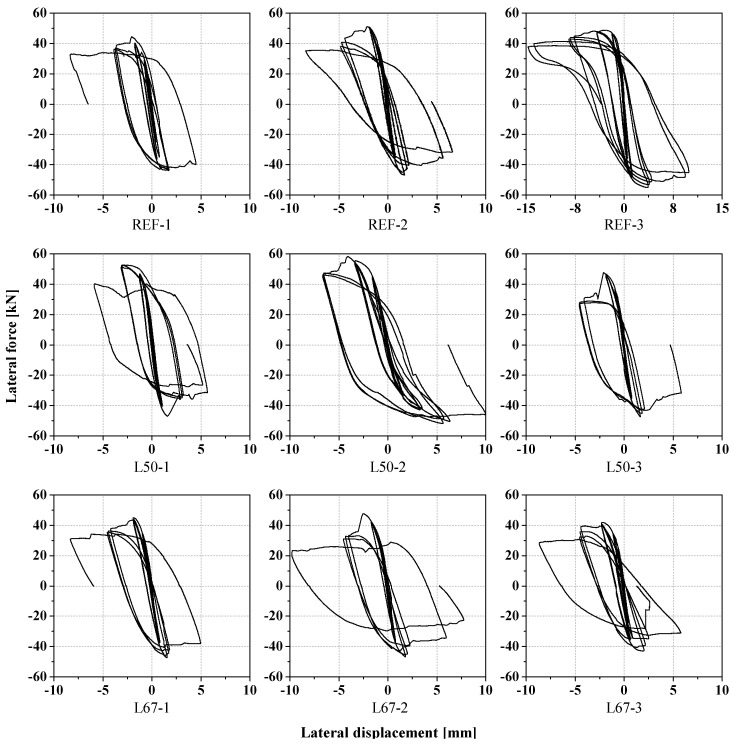
Lateral force against lateral displacement curves obtained for masonry walls with different mortars. Note the different horizontal scale for REF-3.

**Figure 5:**
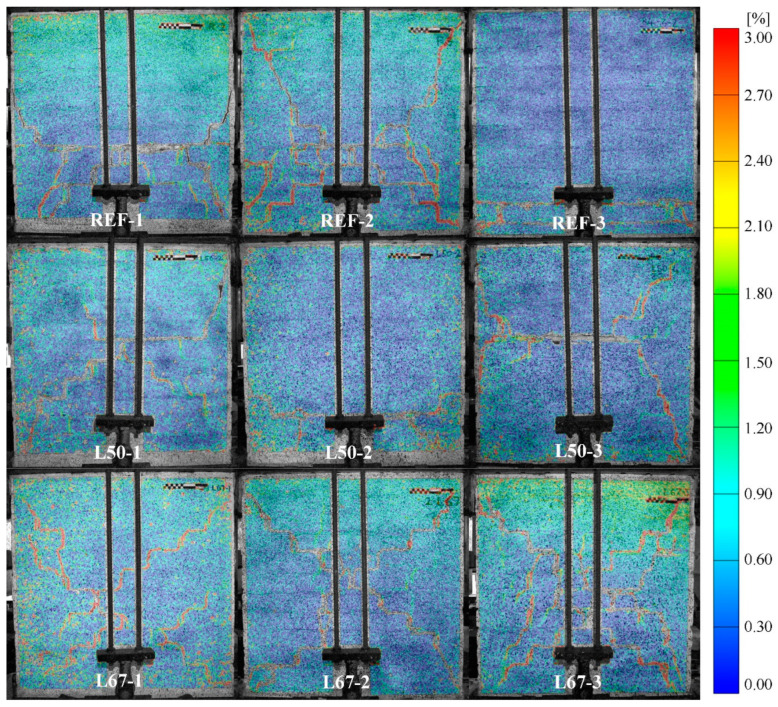
Final crack patterns obtained for masonry walls with different mortars, indicated by maximum principal strains from Digital Image Correlation.

The most common failure mode among the tested specimens was the shear mechanism, characterized by diagonal cracks. The obtained results in terms of lateral force versus lateral displacement are shown in Figure 4. It is noted that the variation in lateral displacements is comparable in most specimens, except for REF-3, which exhibited a different range and shape of the hysteresis diagram. This can be explained by the fact that REF-3 experienced failure due to horizontal sliding through the first two mortar bed joints at the base (see Figure 5). This test was prolonged beyond failure until the measured displacements surpassed the deformations observed in other specimens.

The final crack patterns obtained for the masonry specimens with different mortars are presented in Figure 5. These DIC images correspond to the end of the last loading cycle, with values expressed in terms of maximum principal strains as obtained from the analysis. Overall, the range of principal strains is consistent with reported values in the literature for unreinforced masonry [66]. The REF-1 and REF-2 specimens exhibited a predominant diagonal shear failure mode, with cracks forming in an X-shaped pattern. Moreover, horizontal cracks were observable in these specimens as well, running through a bed joint at approximately mid-height for REF-1 and at a lower position for REF-2. The presence of horizontal cracks suggests some degree of flexural cracking, although the shear mechanism dominated the overall failure. In contrast, REF-3 experienced a distinct sliding failure, characterized by two horizontal cracks at the base. Considering that the level of pre-compression was the same for the other REF specimens, this mode of failure may have been motivated by construction defects that resulted in poor shear bond strength between the mortar and the masonry units. The L50 set exhibited similar diagonal shear failures with X-shaped cracks and horizontal cracks running through mortar bed joints. Specifically, these horizontal cracks appeared close to the center for L50-1 and L50-3, and near the base in L50-2. This indicates a combination of shear and flexural failures, where the horizontal cracks suggest some degree of rocking or flexural response, especially under increased drift levels [38]. The L67 series presented more pronounced diagonal step-like cracks, representative of shear failure mechanisms. These crack patterns, particularly the step-like nature of the diagonal cracks, could be attributed to the reduced tensile and shear strength of the mortar, resulting from the increased lime content, which typically reduces mortar stiffness and bonding strength. Overall, the variations in crack patterns underscore the influence of mortar composition on the failure modes of masonry walls under cyclic loading.

**Figure 6:**
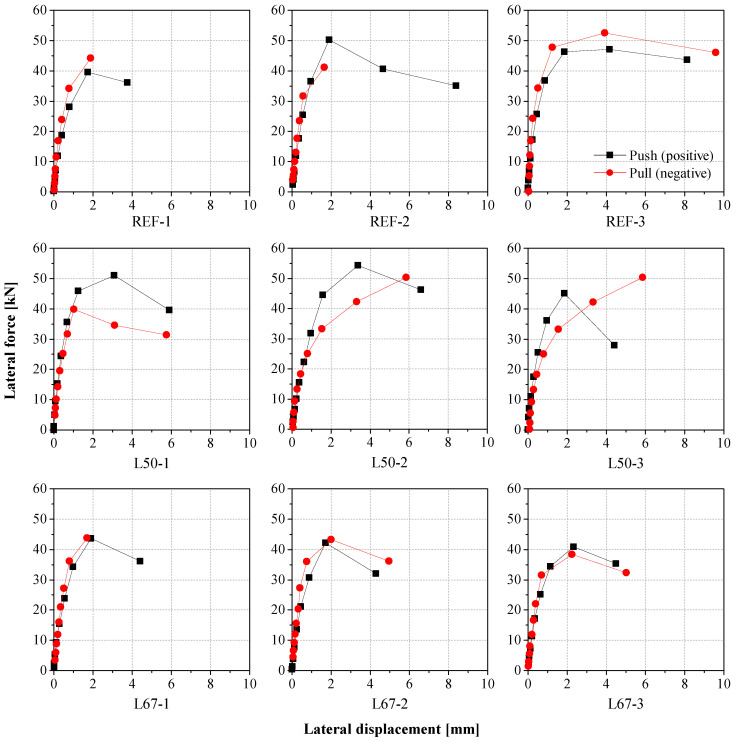
Experimental resistance envelopes obtained for masonry walls with different mortars.

The lateral force-displacement hysteresis diagrams presented in Figure 4 were converted into resistance envelopes in order to assess and compare the responses of the different masonry specimens. These experimental envelopes are presented in Figure 6, for both positive (push of the horizontal actuator) and negative (pull of the actuator) directions. The experimental envelopes were defined for each specimen considering the maximum lateral displacement recorded for each loading cycle, together with the corresponding force [2,70]. Each point on the curve represents the average of the three values measured for each amplitude (three cycles for each level of imposed displacement).

**Figure 7:**
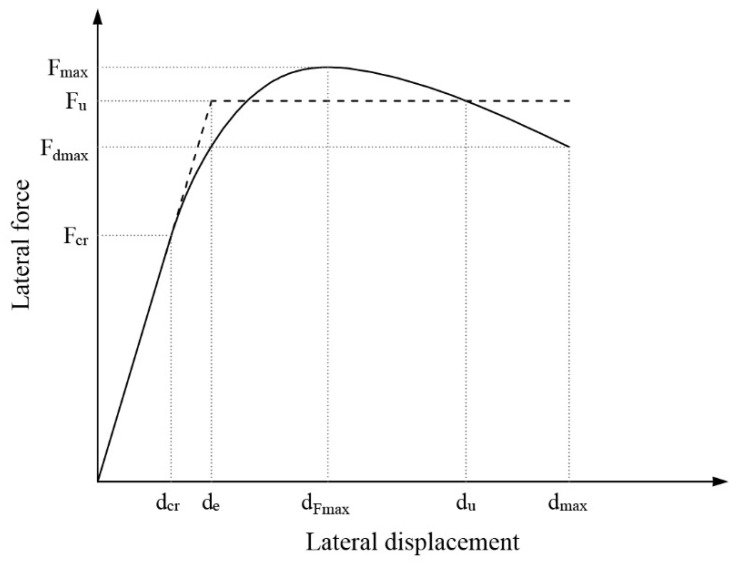
Bilinear fit of experimental resistance envelopes (adapted from Ref. [6]).

A commonly used method in the literature was applied to transform the resistance envelopes into idealized bilinear envelopes, which allows for the evaluation of parameters such as strength, stiffness, and deformation capacity of masonry subjected to in-plane cyclic loading [1,2,6,66,71]. For each curve, three characteristic points were defined in order to assess the performance of the corresponding specimen, as suggested by Tomaževič [6] (see Figure 7).

The first characteristic point on the idealized curve is the crack limit state, determined by displacement $d_{cr}$ [mm] and lateral force $F_{cr}$ [kN]. It represents the onset of significant cracks in the specimen, which translates into a change in the initial slope. The second point is the maximum resistance and is determined by the maximum load $F_{max}$ [kN] attained during the test, and the associated displacement $d_{F,max}$ [mm]. The third point is the ultimate state and is defined by the maximum displacement $d_{max}$ [mm] attained during the test, and its corresponding load $F_{d,max\;}$ [kN]. Since the maximum displacement varied for different specimens depending on the moment when the test protocol was stopped, for consistent quantitative comparison, the maximum displacement $d_{max}$ was defined as the displacement at 85% of the peak load value, following the method used by Deng et al. [26]. It is important to note that for REF-1, the test had to be stopped during post-peak at approximately 91% of its maximum load, as the specimen was about to collapse. Therefore, for uniform comparison, the post-peak segment of the resistance envelope for REF-1 was linearly extrapolated until reaching 85% of its lateral load capacity. Table 6 presents the force and displacement values at these three characteristic points for each specimen.

Considering the idealized envelope, $A_{env}$ [kN·mm] represents the area under the curve and is a measure of the energy dissipation capacity of the system. In turn, $K_{e}$ [kN/mm] is the initial or effective secant stiffness defined by [6]:

(6) $$K_{e}=\frac{F_{cr}}{d_{cr}}$$

In addition, $F_{u}$ [kN] refers to the ultimate resistance, determined as the point that provides equal energy dissipation from both the idealized envelope and the experimental curve [2]. For this term, Tomaževič [6] provides the following expression:

(7) $$F_{u}=K_{e}\left(d_{max}-\sqrt{{d_{max}}^{2}-\frac{2A_{env}}{K_{e}}}\right)$$

Finally, $d_{e}$ [mm] represents the idealized elastic displacement, i.e., the point where the effective stiffness intersects with the ultimate resistance, and can be calculated as [6]:

(8) $$d_{e}=\frac{F_{u}}{K_{e}}$$

Thus, the experimental envelopes obtained for each specimen and Equations (6)–(8) were used to calculate the main parameters associated with the idealized bilinear envelopes, as presented in Table 7. First, ${µ}_{p}$ was defined as the ratio $d_{u}/d_{e}$, accounting only for post-peak behavior [2]. In order to understand ductility before reaching maximum capacity, another parameter, ${µ}_{e}$, was introduced. This parameter is defined as the ratio $d_{F,max}/d_{cr}$ and represents the deformation capacity of the specimen between the appearance of the first significant cracks and its maximum lateral capacity. Finally, the secant stiffness at maximum capacity, $K_{max}$ [kN/mm], was defined as the ratio of $F_{max}/d_{F,max}$.

From Table 6 and Table 7, it can be observed that the overall range of values obtained for various parameters is consistent among different specimens. To evaluate the impact of the type of mortar on the response, it is necessary to examine the mean values across specimens with the same mortar type, as detailed in Table 8. Notably, since REF-3 failed due to sliding, the deformations in this specimen were much larger than in other specimens. For consistency, REF-3 has not been considered in any averaged value related to deformation. Moreover, the displacements associated with crack limit state, $d_{cr}$, and maximum capacity, $d_{F,\max}$, have been expressed as drifts, calculated as lateral deformation with respect to the height of the specimen, in percentage.

The analysis of the averaged values reveals some interesting observations. Firstly, L50 exhibits slightly higher maximum lateral capacity, $F_{max}$, compared to REF, followed by L67. This result is unexpected when compared to the findings from previous tests on compressive, flexural, and bond strength capacity [72]. Moreover, L50 also shows the highest crack limit state, $F_{cr}$, with a 33% higher value compared to REF, while REF and L67 show similar values. With regard to the ratio $F_{cr}/F_{max}$, L50 shows the first significant cracks at 62% of its maximum lateral capacity, followed by L67 at 55%, and REF at 51%. It is noted that the observed lateral strength capacity trends do not align with the trends obtained for strength at the mortar level, where the maximum is REF, followed by L50, and L67 (Table 4). In terms of initial secant stiffness, $K_{e}$, and maximum secant stiffness, $K_{max}$, REF exhibits the highest values. Overall, the findings suggest that L50 provides higher lateral capacity while exhibiting slightly lower secant stiffness values in contrast to the other two mortar mixes.

Notably, the ratio $F_{u}/F_{max}$ is consistent across all mortar mixes, with a value of 91%. This observation is in line with findings from other experimental research works on more than 60 masonry walls [6]. On the other hand, the ratio $F_{cr}/F_{max}$ ranged between 0.5 and 0.6. These ratios are slightly lower than other values reported in the literature, between 0.6 and 0.8, or the average 0.7 commonly considered in practical applications [1,18]. Nonetheless, considering the work of Vasconcelos [2], the calculation of ratios $F_{cr}/F_{max}$ tend to be around 0.5 as well, and values within this same range can be found in other works in the literature [73].

The trends for drift capacity corresponding to both crack limit state and maximum resistance are similar to that observed for the ratio $F_{cr}/F_{max}$. Specifically, L50 exhibits the highest drift capacity, followed by L67, and finally REF. Regarding the drift capacity at the ultimate state, L50 still shows the highest value, now followed by REF, and L67 last. Overall, the range of values for the ultimate drift capacity, with an average of 0.4%, is in line with the experimental results reported by Magenes & Calvi [18] for similar unreinforced brick masonry walls, around 0.5%. Concerning the post-peak ductility, $\mu_{p}$, REF has the best performance, followed by L50 and L67, with comparable values. Conversely, for pre-peak ductility, $\mu_{e}$, REF and L50 exhibit comparable results, followed by L67 with slightly lower values.

Following the work of Abrams [74], the drift capacity of unreinforced masonry walls can be analyzed through three performance levels, namely Immediate Occupancy (IO), Life Safety (LS), and Collapse Prevention (CP). IO corresponds to the onset of shear cracks, usually at about 90% of the maximum load, $F_{max}$ [2,18]. In this work, since $F_{u}\approx$ 0.9·$F_{max}$, the idealized elastic displacement, $d_{e}$, was used for the evaluation of IO (Equation (8)). LS corresponds to the drift at maximum lateral capacity, indicated by $d_{F,max}$, and CP corresponds to the ultimate drift or collapse state of the structure, defined by $d_{max}$ [74]. The values for the three performance-based drift levels determined for the tested masonry specimens in this study are shown in Table 9, with REF-3 excluded from the averaged values as it failed through a sliding mechanism. An average value of IO around 0.10% was obtained for all cases, which is consistent with the reported values in the literature for unreinforced masonry buildings [75]. Similarly, only slight differences were found for LS and CP levels for all the mortars, which ranged from 0.20% to 0.31%, and from 0.45% to 0.54%, respectively.

**Figure 8:**
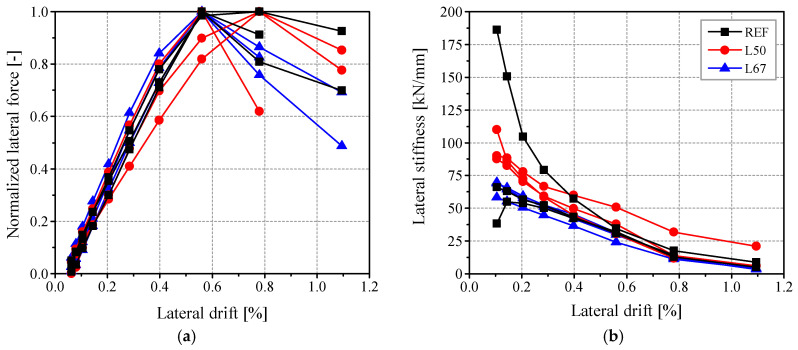
Performance of the tested masonry walls with respect to lateral drift: (**a**) normalized lateral resistance; (**b**) stiffness degradation.

Figure 8 presents the performance of the different masonry specimens in terms of drift capacity. In particular, Figure 8a shows the normalized lateral forces (calculated with respect to maximum load) against lateral drift. Considering these normalized experimental envelopes, three specimens, namely REF-3 (which failed by sliding mechanism), L50-1, and L50-2, exhibited significant lateral strength capacity at a drift of 0.8%. The other specimens behaved similarly, reaching their maximum capacity at a drift of around 0.6%. In turn, Figure 8b illustrates the degradation of lateral stiffness with respect to the drift, which serves as an indication of the propagation of damage in the specimen [26]. It is noted that the secant stiffness was calculated for each cycle as the slope of the line connecting the minimum and maximum strength values observed in that cycle [2]. In terms of stiffness degradation, most specimens exhibited similar behavior, except for L50-2, which displayed slightly higher stiffness at 1.1% drift. Another notable difference was observed for REF-3 (sliding failure mechanism), which initially showed the highest stiffness value, but ultimately reached a stiffness range comparable to the other specimens.

The energy dissipation capacity is another important parameter for the analysis of masonry structures subjected to seismic loads, as it contributes to the mitigation of response amplitude, thus reducing the demands on ductility [4]. In this study, the energy dissipated by the masonry specimens was calculated from the area under the force-displacement hysteresis loop for each cycle. It is noted that global energy dissipation depends on several factors, such as the applied compressive stress [2]. For quantitative comparison, Shing et al. [4] proposed a cumulative normalized energy parameter, $E_{N}$ [-], which employs the idealized bilinear envelope and its associated parameters to normalize the amount of dissipated energy. In particular, the equation proposed by Vasconcelos [2] was used to calculate this term:

(9) $$E_{N}=\frac{1}{F_{u}d_{e}}\overset{n}{\underset{i=1}{\sum }}E_{i,\;dissipated}$$

where n is the number of load cycles.

**Figure 9:**
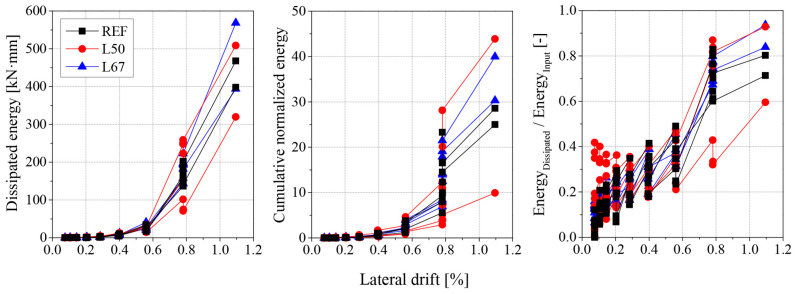
Dissipated energy against lateral drift for masonry walls with different mortars.

Additionally, the energy input, or energy required to apply lateral deformation on the specimens, was compared against the total energy dissipated by the specimens [38]. In this context, the energy input was determined as the area under the hysteresis loop of each cycle. Figure 9 shows the dissipated energy, the cumulative normalized energy, $E_{N}$, and the relation of dissipated energy to energy input for each specimen. There is no clear trend in terms of energy dissipation, both in absolute and normalized terms, as all specimens behave similarly. However, the ratio of dissipated energy to energy input tends to increase as lateral drift increases or with increasing number of cycles, approaching but never reaching one. It is evident that the development of damage increases the value of this ratio, with a noticeable jump between 0.6% and 0.8% lateral drift, which coincides with the variation in lateral capacity (90% to 100%, or $F_{u}$ to $F_{max}$) as observed in Figure 8a, indicating the appearance of shear cracks.

It is evident that the determination of in-plane shear strength of masonry is a complex task that requires significant time and resources. In this sense, various simplified formulations have been proposed by researchers and incorporated into masonry design codes for practical use. Thus, the applicability of these formulations was assessed by comparing their predictions with the experimental values obtained in this study. Balasubramanian et al. [11] provided a comprehensive review of the different formulations for the three failure modes related to in-plane shear response of unreinforced masonry, namely sliding, diagonal shear, and rocking. The relevant formulations for diagonal shear cracking, which is the failure mode of interest for this study, are recalled herein for the sake of clarity.

Eurocode 6 [17] proposes the following expression to determine the shear capacity:

(10) $$V_{d}=\frac{f_{vk}\;t\;l}{\gamma_{m}}\;\;\;\;\;\mathrm{where}\;f_{vk}=f_{vk0}+\mu \;\sigma \;\;\;\;\;\;\;$$

where $V_{d}$ [N] is the maximum lateral capacity, $f_{vk}$ [Pa] represents the characteristic shear strength of masonry, which depends on the initial shear strength, $f_{vk0}$ [Pa], the friction coefficient, μ (typically 0.4), and the perpendicular compressive stress, σ [Pa], t [m] and l [m] are the thickness and length of the specimen, respectively, and $\gamma_{m}$ [-] is the safety coefficient for the material.

Secondly, Magenes & Calvi [18] proposed the following analytical expression:

(11) $$V_{d}=t\;l\;\tau_{u}\;\;\;\;\;\;\mathrm{where}\;\tau_{u}=\min\left(\tau_{c},\;\tau_{w}\right)\;\;\;\;$$

$$\tau_{c}=\frac{1.5\;c+\mu \;\sigma }{1+3\;c\;\alpha_{V}/\sigma }\;\;\;\;\;\;\mathrm{where}\;\alpha_{v}=h_{o}/l$$

$$\tau_{w}=\frac{c+\mu \;\sigma }{1+\alpha_{V}}$$

where $\tau_{u}$ [Pa] is the maximum shear strength, defined as the minimum value of $\tau_{c}$ [Pa] or $\tau_{w}$ [Pa], calculated for the cracked section or the whole section, respectively, c [Pa] is the cohesion, and $\alpha_{V}$ is the geometrical shear ratio defined by the relation between the effective height, $h_{0}$ [m], and the length of the wall, [m].

Finally, Vasconcelos [2] proposed the following expression:

(12) $$F_{f}=\frac{t\;l^{2}}{6\;h_{o}}\left(\sigma +f_{tj}\right)\;$$

where $F_{f}$ [N] is the horizontal load associated with the onset of flexural cracks, caused by the loss of adhesion between units and mortar [2], and $f_{tj}$ [Pa] represents the tensile strength of the mortar bed joint. Considering that the formation of cracks results in a change in stiffness, $F_{f}$ would be analogous to the definition of $F_{cr}$ in idealized bilinear envelopes, provided that a linear distribution of stresses is assumed [2].

The comparison between the values obtained from the analytical expressions in Equations (10)–(12) and the values obtained from experiments on the masonry specimens with different mortars is presented in Table 10. In general, the approach proposed by Magenes & Calvi [18] (Equation (11)) provides a more accurate estimation of the maximum lateral capacity compared to Eurocode 6 [17] (Equation (10)). Additionally, the expression proposed by Vasconcelos [2] (Equation (12)) for the onset of damage generally results in lower estimations than the actual experimental data.

## 5. Conclusions

This study presents a systematic experimental investigation into the influence of varying lime content in cement-lime mortars on the in-plane cyclic behavior of unreinforced masonry walls. To the authors’ knowledge, this is a novelty in the scientific literature. In particular, the structural performance of single-leaf brick masonry walls under in-plane cyclic shear loading was evaluated in terms of energy dissipation, stiffness degradation, and drift capacity. The response of the tested masonry walls showed no distinct trends related to the addition of lime to the blended mixes or the compressive strength of the resulting mortars. The specimens were initially subjected to a vertical compression of 0.78 MPa, and then horizontal lateral displacements were imposed to obtain force-displacement hysteresis diagrams. These diagrams were employed to construct idealized bilinear envelopes, and various parameters were extracted and compared to evaluate the performance of the different mortars.

The results showed that the maximum lateral capacity was the highest for the walls with mortar L50 with a value of 0.55 MPa, followed by REF with 0.50 MPa, and L67 with 0.46 MPa. The lateral forces ranged between 40 kN and 50 kN. Conversely, the value of secant stiffness at maximum load for mortar L50 was the lowest with 19 kN/mm, followed by L67 with 22 kN/mm, and REF with 25 kN/mm. In turn, the drift percentage at cracking was again highest for L50 with 0.08%, followed by L67 with 0.06%, and REF with 0.05%. Likewise, the drift percentage at peak capacity maintained the same trend, i.e., highest for L50 with 0.31%, followed by L67 with 0.22%, and REF mortar with 0.20%. Similarly, the ratio $F_{cr}/F_{max}$, which indicates the percentage of maximum capacity at which masonry begins to crack, followed the same order, being L50 the highest with 0.60, followed by L67 with 0.55, and REF with 0.51. Finally, the ratio $F_{u}/F_{max}$ was similar for all specimens, approximately 0.91. Stiffness degradation and energy dissipation were comparable for all cases as well.

It was observed that the in-plane shear capacity of masonry is overestimated by Eurocode 6 [17], whereas the approach by Magenes & Calvi [18] provides a better estimate of the lateral capacity, with less than 15% difference between analytic and experimental results. Notably, the experimental values obtained for mortar REF were lower than expected, although a clear explanation for these results could not be determined.

From a practical standpoint, the results of this study suggest that replacing a portion of cement with lime in masonry mortars does not compromise the mechanical performance of masonry walls subjected to in-plane cyclic loads. This has direct implications for the construction industry, particularly in promoting the use of more sustainable building materials. The environmental impact of lime is generally lower than that of cement, which contributes to reducing the carbon footprint of masonry construction. Thus, adopting lime-rich mortars in unreinforced masonry could be a viable strategy for achieving sustainability goals in building practices, aligning with emerging trends in sustainable construction and the United Nations’ 2030 Agenda for Sustainable Development.

It must be noted that the scope of this study is limited by its focus on varying only the mechanical properties of the mortar while holding constant other influential parameters, such as constraint conditions, vertical compression level, wall slenderness, and masonry texture. These factors can also affect the in-plane cyclic behavior of masonry walls. The results obtained here might differ if any of these other parameters were varied. Thus, further research is necessary to explore the interrelations between these parameters and to assess how the combined effects might influence the cyclic performance of unreinforced masonry walls.

## Figures and Tables

**Table 1:** Chemical composition of binders (CEM I-42.5 R and CL 90-S) as provided by the manufacturers.

Material	Al_2_O_3_ [%]	MgO [%]	Fe_2_O_3_ [%]	SO_3_ [%]	SiO_2_ [%]	K_2_O [%]	CaO [%]	LOI [%]
Cement	4.27	1.75	3.20	3.05	20.55	0.77	63.40	2.05
Lime	0.06	0.68	0.05	0.197	0.12	0.013	74.35	25.00

**Table 2:** Mechanical characterization of bricks (CoV in % between parentheses).

Compressive Strength [MPa]		Flexural Strength [MPa]	Young’s Modulus [GPa]	Water Absorption [%]	IRA [kg/(m^2^·min)]
Complete Brick	Cubic Specimen				
22.03 (22.7)	21.15 (13.7)	5.41 (21.0)	4.9 (15.7)	10.3 (7.6)	3.55 (15.6)

**Table 3:** Composition of the mortars used in masonry.

Cement:Lime:Sand (by Volume)	Nomenclature	Cement [kg]	Lime [kg]	Aggregate [kg]		w/b Ratio (by Volume)	w/b Ratio (by Weight)
				Sand	Filler		
1:0:5	REF	233.5	0.0	1743.6	206.4	1.20	1.12
1:1:6	L50	192.6	73.4	1726.1	204.4	1.09	0.70
1:2:9	L67	128.4	97.9	1726.1	204.4	1.30	0.71

**Table 4:** Comparison of mechanical properties of the mortars used in masonry, cured in situ and in standard conditions, tested at 28 days and 90 days of age (CoV in % between parentheses).

Mortar Type	Compressive Strength [MPa]				Flexural Strength [MPa]				E-Modulus [GPa]	
	Standard		In Situ		Standard		In Situ		Standard	In Situ
	$f_{c-28}$	$f_{c-90}$	$f_{c-28}$	$f_{c-90}$	$f_{b-28}$	$f_{b-90}$	$f_{b-28}$	$f_{b-90}$	$E_{\;90}$	$E_{\;90}$
REF	10.27	11.21	10.88	12.08	3.14	3.53	3.04	3.78	19.47	15.21
	(7.3)	(2.7)	(8.9)	(6.0)	(2.4)	(7.1)	(1.0)	(11.3)	(10.5)	(5.1)
L50	9.35	9.28	9.75	10.07	3.93	3.42	2.99	3.55	14.86	15.97
	(5.1)	(5.7)	(7.6)	(8.5)	(0.7)	(3.5)	(9.3)	(7.8)	(2.2)	(17.5)
L67	4.35	4.69	4.12	5.30	1.88	1.88	1.57	1.95	8.54	7.90
	(10.8)	(2.1)	(3.4)	(5.2)	(5.0)	(4.5)	(3.5)	(2.1)	(1.2)	(5.3)

**Table 5:** Horizontal deformations imposed on masonry specimens for in-plane cyclic loading test.

**Amplitude [mm]**	0.49	0.68	0.95	1.30	1.84	2.57	3.59	5.02	7.02	9.83	13.77
**Drift [%]**	0.05	0.08	0.11	0.14	0.20	0.29	0.40	0.56	0.78	1.09	1.53
**Speed [m/s]**	0.03	0.03	0.03	0.05	0.05	0.05	0.07	0.07	0.07	0.07	0.10

**Table 6:** Limit states from experimental resistance envelopes, namely crack limit ($F_{cr},d_{cr}$), maximum resistance ($F_{max},d_{F,max}$), and ultimate state ($F_{d,max},d_{max}$).

Specimen	$F_{cr}$ [kN]	$d_{cr}\;$[mm]	$F_{max}$ [kN]	$d_{max}$ [mm]	$F_{d,max}$ [kN]	$d_{F,max}\;$[mm]
REF-1	18.8	0.40	39.5	1.74	33.6	5.19
REF-2	25.4	0.55	50.2	1.89	42.7	4.06
REF-3	25.7	0.44	47.1	4.17	40.0	12.21
L50-1	35.6	0.67	51.0	3.09	43.4	4.97
L50-2	31.8	0.96	54.3	3.37	46.1	6.64
L50-3	25.6	0.51	45.2	1.85	38.4	2.86
L67-1	23.9	0.54	43.7	1.91	37.2	4.06
L67-2	21.0	0.46	42.2	1.72	35.9	3.31
L67-3	25.2	0.63	40.9	2.33	34.8	4.71

**Table 7:** Parameters used for the bilinear fit of experimental resistance envelopes.

Specimen	$F_{u}$ [kN]	$d_{u}$ [mm]	$d_{e}$ [mm]	$K_{e}$ [kN/mm]	$K_{max}$ [kN/mm]	FcrFmax	FuFmax	${µ}_{e}$	${µ}_{p}$
REF-1	35.7	3.95	0.77	46.3	22.7	0.47	0.90	4.3	5.1
REF-2	45.2	3.35	0.99	45.9	26.6	0.51	0.90	3.4	3.4
REF-3	43.9	7.80	0.75	58.3	11.3	0.55	0.93	9.4	10.4
L50-1	47.8	3.90	0.90	53.0	16.5	0.70	0.94	4.6	4.3
L50-2	49.7	5.15	1.50	33.1	16.1	0.59	0.92	3.5	3.4
L50-3	40.8	2.48	0.81	50.3	24.4	0.57	0.90	3.6	3.1
L67-1	39.8	3.20	0.90	44.1	22.9	0.55	0.91	3.5	3.6
L67-2	37.6	2.85	0.82	45.9	24.6	0.50	0.89	3.7	3.5
L67-3	37.6	3.65	0.94	40.1	17.6	0.61	0.92	3.7	3.9

**Table 8:** Average values of parameters obtained from in-plane cyclic loading tests according to the type of mortar used in masonry specimens (CoV in % between parentheses).

Mortar Type	$F_{max}$ [kN]	$F_{cr}$ [kN]	$K_{e}$ [kN/mm]	$K_{max}$ [kN/mm]	$Drift_{cr}$ [%]	$Drift_{F,max}$ [%]	$Drift_{u}$ [%]	FcrFmax	FuFmax	${µ}_{e}$	${µ}_{p}$
REF	45.6 (12.0)	23.3 (16.9)	46.1 (15.2)	24.6 (11.0)	0.05 (22.0)	0.20 (5.9)	0.41 (11.6)	0.51 (7.1)	0.91 (1.9)	3.9 (16.3)	4.3 (28.7)
L50	50.1 (9.2)	31.0 (16.2)	45.5 (23.7)	19.0 (24.7)	0.08 (31.9)	0.31 (29.3)	0.43 (34.8)	0.62 (11.4)	0.92 (1.8)	3.9 (15.3)	3.6 (18.1)
L67	42.3 (3.3)	23.4 (9.0)	43.4 (6.8)	21.7 (16.9)	0.06 (15.5)	0.22 (15.8)	0.36 (12.4)	0.55 (10.5)	0.91 (1.5)	3.7 (3.3)	3.6 (6.1)

**Table 9:** Performance-based drift levels for masonry walls with different mortars (CoV in % between parentheses).

Performance	IO	LS	CP	IO-Avg	LS-Avg	CP-Avg
Specimen	$Drift_{e}$ [%]	$Drift_{F,max}$ [%]	$Drift_{max}$ [%]	$Drift_{e}-Avg$ [%]	$Drift_{F_{max}}-Avg$ [%]	$Drift_{max}-Avg$ [%]
REF-1	0.09	0.19	0.58			
REF-2	0.11	0.21	0.45	0.10 (17.3)	0.20 (5.9)	0.51 (17.3)
REF-3	0.08	0.46	1.36			
L50-1	0.10	0.34	0.55			
L50-2	0.17	0.37	0.74	0.12 (35.0)	0.31 (29.3)	0.54 (39.3)
L50-3	0.09	0.21	0.32			
L67-1	0.10	0.21	0.45			
L67-2	0.09	0.19	0.37	0.10 (6.8)	0.22 (15.8)	0.45 (7.3)
L67-3	0.10	0.26	0.52			

**Table 10:** Analytical and experimental in-plane shear capacity of masonry walls with different mortars (CoV in % between parentheses).

Mortar Type			$F_{\max}$ [kN]				$F_{cr}$ [kN]	
	Experimental	EC-6 [17]	Diff [%]	Magenes & Calvi [18]	Diff [%]	Experimental	Vasconcelos [2]	Diff [%]
REF	45.6 (12.0)	58.9	29.2	52.7	15.5	23.3 (16.9)	23.0	−1.5
L50	50.1 (9.2)	49.8	−0.8	53.4	6.4	31.0 (16.2)	23.4	−24.5
L67	42.3 (3.3)	50.7	19.9	41.4	−2.0	23.4 (9.0)	20.7	−11.6

## Data Availability

The original contributions presented in the study are included in the article, further inquiries can be directed to the corresponding author.

## Notation

$A_{env}$	Area under resistance envelope [N·m]
c	Cohesion [Pa]
CoV	Coefficient of variation [%]
$d_{cr}$	Crack limit lateral displacement [m]
$d_{e}$	Idealized elastic displacement [m]
$d_{F,max}$	Displacement at maximum lateral force [N]
$d_{max}$	Maximum lateral displacement [m]
$d_{u}$	Displacement at ultimate lateral force [m]
E	Elastic modulus [Pa]
$E_{N}$	Cumulative normalized energy [–]
$F_{cr}$	Crack limit lateral force [N]
$F_{d,max}$	Force at maximum lateral displacement [N]
$F_{f}$	Flexural cracking onset lateral force [N]
$F_{\max}$	Maximum lateral force [N]
$F_{u}$	Ultimate lateral force [N]
$f_{b}$	Flexural strength [Pa]
$f_{c}$	Compressive strength [Pa]
$f_{t}$	Tensile strength [Pa]
$f_{tb}$	Tensile strength of brick [Pa]
$f_{tj}$	Tensile strength of mortar bed joint [Pa]
$f_{vk}$	Characteristic shear strength [Pa]
$f_{vk0}$	Initial shear strength [Pa]
$h_{0}$	Height [m]
j	Rectangular compressed section distribution factor [–]
$K_{e}$	Effective secant stiffness [N/m]
$K_{max}$	Secant stiffness at maximum capacity [N/m]
l	Length [m]
n	Number of load cycles [–]
r	Height/length ratio factor [–]
t	Thickness [m]
$V_{d}$	Design shear force (maximum lateral capacity) [N]
w/b	Water to binder ratio [–]
$\alpha_{V}$	Height-to-length ratio
$\gamma_{m}$	Material safety coefficient [–]
μ	Friction coefficient
$\mu_{e}$	Maximum force to crack limit displacement ratio [–]
$\mu_{p}$	Ultimate to idealized displacement ratio [–]
σ	Compressive stress [Pa]
τ	Shear stress [Pa]
$\tau_{c}$	Shear stress relevant to the cracked section [Pa]
$\tau_{max}$	Shear stress at maximum lateral load capacity [Pa]
$\tau_{w}$	Shear stress relative to the whole section [Pa]
